# Vive la Persistence: Engineering Human Microbiomes in the 21st Century

**DOI:** 10.1128/mSystems.00166-17

**Published:** 2018-04-10

**Authors:** Katrine L. Whiteson

**Affiliations:** a Department of Molecular Biology & Biochemistry, University of California, Irvine, California, USA

**Keywords:** metabolomics, microbiome, phage therapy

## Abstract

I imagine a future in which children grow up with healthy microbial communities. Engineering human microbiomes might actually be achievable in the near future, as we enter an era of hunting for human-adapted bacterial strains and phages. Furthermore, breath metabolites could allow us to track whether a probiotic colonizes persistently or a phage has knocked down a microbe of interest. Recent successes with probiotics, such as bifidobacteria that can break down human milk oligosaccharides, are making a future in which infants are intentionally colonized with health-promoting strains seem less unlikely. Viruses that infect bacteria, bacteriophages, are also important for human health both because of their role in the human microbiome and because of their potential for use in phage therapy. Monitoring the outcome of microbiome-focused interventions with breath volatile sampling is also on the horizon, which could mean real-time tracking of microbial metabolite production. Studies of early life during microbiome assembly, when the potential for effective interventions to reduce disease risk is greatest, are essential.

## Abstract (precis)

I imagine a future in which children grow up with healthy microbial communities. Engineering human microbiomes might actually be achievable in the near future, as we enter an era of hunting for human-adapted bacterial strains and phages. Furthermore, breath metabolites could allow us to track whether a probiotic colonizes persistently or a phage has knocked down a microbe of interest.

## PERSPECTIVE

The quest to ensure that all humans have access to a healthy microbiome first requires us to define what is meant by “healthy.” This is especially important in the face of a 20th-century increase in the rates of autoimmune disease, allergies, obesity, and other conditions that we now understand are likely tied to changes in microbial exposure (1). Even after more than 10 years of intense study and with incredible advances in access to sequence data, we are still far from understanding the ideal composition and activity of human microbial communities (2). Despite this, there are a few areas where we do know enough to make tangible, practical progress. On several fronts, exciting and implementable developments are moving more rapidly than I had once expected. In this perspective, I focus on the following three questions. (i) Can we shape infant microbiomes and help shape healthy immune development? (ii) Can we specifically target bacterial strains to combat infection and engineer microbiomes? (iii) How can we monitor successful microbiome engineering?

### Can we shape infant microbiomes and help shape healthy immune development?

If I had written this article a few years ago, I would have argued that the relatively modest goal of understanding what the infant microbial community should look like would still be several years away, and that more ambitious hopes of shaping those communities would be much further in the future. But more progress has been made than I anticipated. Several studies of infants in the developed world show that infants initially have diverse gut microbiomes (3, 4) largely derived from their mother and a bit later, as they are weaned, starting to come from the environment (5). However, bacteria that are able to access the 15% of energy in breast milk in the form of human milk oligosaccharides not accessible to human digestion (e.g., Bifidobacterium longum subsp. infantis) may have important colonization advantages along with health advantages (6, 7). In a cohort of roughly 70 mothers and breastfed infants, half of the infants were given a B. longum subsp. infantis supplement and developed gut microbiomes dominated by B. longum subsp. infantis along with marked decreases in fecal milk oligosaccharides, increases in low-pH fermentation products, decreases in bacterial toxins, and a reduction in the number of stools per day (8). The short-term results can only demonstrate what the company (Evolve Biosystems) carefully claims, i.e., that taking the B. longum subsp. infantis supplement leads to colonization of a breastfeeding infant. The potential implications are much grander, although we will need much more research to confirm this: colonization of the infant gut by bifidobacteria during the first critical months of life during immune development could ameliorate the continuing dramatic increases in autoimmune and allergic disease (1). In the summer of 2017, a study was published of a trial of a probiotic *Lactobacillus* chosen for its ability to colonize infant guts for up to 4 months; among roughly 4,000 infants in rural India, the incidence of sepsis was reduced by approximately 40% (9) (microbiome-related data from the study are not yet available). Critically, the strain used was adapted to live in humans, rather than in food, and was chosen for its ability to colonize the infant gut persistently when relevant oligosaccharides are available. Microbiome researchers have come to expect probiotics, often chosen for ease of production rather than the ability to colonize a human, to only transiently affect human microbial communities. However, we can imagine a future where human-adapted strains known to promote healthy immune development are given to babies during critical developmental windows. It is a big responsibility to choose the bacterial strains that recolonize humanity after a century of sanitation, antibiotics, C-section, and formula feeding. Uncomfortably, human gut strains need to come from human guts; this means that well-meaning attempts such as seeding babies with maternal vaginal secretions and eating yogurt are unlikely to be effective, as they provide exposure to yogurt or vaginally adapted strains, rather than gut-adapted strains that can colonize a human and have been passed from parents to children and within close-knit groups of people in earlier eras with less sanitation. This is an important future area for probiotic startups looking to enter the billion-dollar probiotic industry. The results of trials with bifidobacteria and other human gut-adapted strains that are able to effectively colonize are eagerly awaited.

### Can we specifically target bacterial strains to combat infection and engineer microbiomes?

This year, there were several successful cases in the United States in which the FDA gave a compassionate-use exception for phage therapy as treatment for an infection, including one in which a University of California San Diego professor emerged from a 9-month coma induced by an Acinetobacter baumanni infection, in part because his wife remembered learning about phage therapy in college (10). The successful examples of probiotic incorporation into infant gut communities and phage therapy in the United States are significant together because phage could be critical for shaping microbial communities. Phage often have narrow host ranges, targeting specific bacterial hosts, which would avoid the decimation of the background microbiota that occurs with many antibiotics (11). The human gut harbors at least as many phage as bacteria; phage diversity and the immune response are both largely uncharacterized (12), and much of the diversity that makes each human gut microbiome unique is imparted as a result of arms races between phages and their bacterial hosts (13, 14). In a recent study of preterm infant gut microbial community composition, all of the babies were given antibiotics and *Enterococcus* spp. were the most abundant bacteria (15). This motivated us to study the interaction of *Enterococcus* spp. and their phage in coevolution experiments, both to understand the diversity-generating interactions between bacteria and their phage that are occurring in all of us and to understand how these dynamics might play out if we wanted to use phage to open up niche space and leave room for probiotic strains such as Evolve Biosystems’ bifidobacteria.

Phage-bacterium arms race dynamics do lead to the selection of phage-resistant strains of bacteria. Using this to our advantage, we can unleash phages that use multidrug-resistant (MDR) pumps as their bacterial host entry point not only to reduce the bacterial load of an infection but also to select for bacteria that lack the MDR and are therefore not susceptible to the phage, leading to renewed antibiotic sensitivity (16). In addition, there are situations where a phage-led reduction in bacterial counts could enable the immune system to clear the infection, in a kind of one-two punch. Furthermore, treating with several phages together in a cocktail could avoid selection for phage-resistant bacteria, especially when resistance comes at a high fitness cost.

### How can we monitor successful microbiome engineering?

Whether next-generation probiotics or phages are the tools at hand, real-time monitoring of microbiome engineering will enable clinicians and patients to track their activity. As many as half of the thousands of small-molecule metabolites in a given plasma or fecal sample are likely to be produced or altered by microbes (17). Microbial metabolites are indicators of particular microbes and their local conditions, and they also interact with other microbes and host cells as nutrients and signals. For example, 2,3-butanedione is a microbial fermentation product universally detected in human breath (18) but sometimes attributed to dairy products in the diet rather than the densely colonized oral cavity with microbes that can produce it. We found that the toxic, butter-flavoring microbial fermentation product 2,3-butanedione is elevated in the breath of cystic fibrosis (CF) patients (19) and could be produced by *Streptococcus* and *Rothia* bacteria on the basis of metagenomic sequences. We then cultured isolates from CF patients and fed them ^13^C-labeled glucose to confirm that the strains we suspected were indeed producing ^13^C-labeled 2,3-butanedione (20). Breath molecules could enable easier real-time testing than fecal samples, so that microbial metabolic products could be tracked through time following food intake and other actions that influence metabolite production on a time scale that is not possible when relying on fecal sample collection. The impact of microbes on breath molecule profiles has been appreciated for decades; Linus Pauling wrote a 1971 paper in the *Proceedings of the National Academy of Sciences of the United States of America* demonstrating that people eating a diet consisting of molecules <100 Da in size, essentially starving the gut microbes, had breath molecule profiles that were more similar to one another (21). A future where infection conditions could be monitored by breath testing might enable earlier and more specific antibiotic treatments.

**FIG 1:**
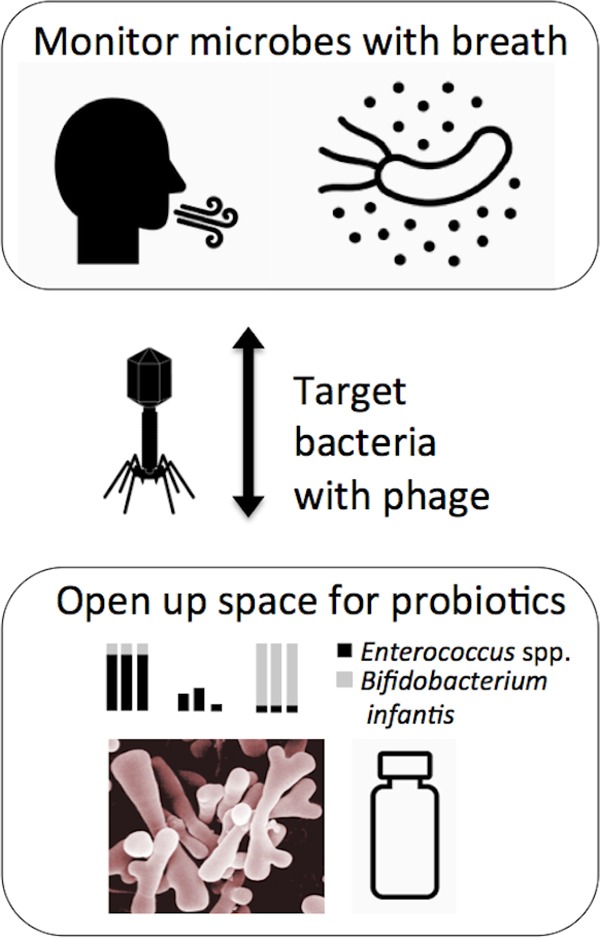
Coupling of breath metabolomics with microbiome engineering. The ability to monitor microbial activity in an accessible and comprehensive sample such as breath could help confirm that a phage has succeeded in knocking down a targeted bacterium or that a probiotic strain is persistently colonizing.

Because each person is unique, a critical aspect of our strategy is to study microbes and metabolites from longitudinal human samples so that we can use a person’s own baseline as a control. Using breath or other accessible metabolomic profiling to track microbial activity would be especially powerful when coupled with tools to specifically modulate the composition of the microbial community (Fig. 1).

Overall, in the next ~5 years, while I expect that we will continue to establish what a healthy microbiome is, I also expect to see real-world examples of microbiome engineering and the ability to monitor this success, perhaps by breath metabolomics. I look forward to seeing human-adapted strains and the dietary molecules that sustain them implemented as effective, persistent colonizers, and phages unleashed against specific bacterial hosts.
